# A HIF-independent, CD133-mediated mechanism of cisplatin resistance in glioblastoma cells

**DOI:** 10.1007/s13402-018-0374-8

**Published:** 2018-02-28

**Authors:** Eroje M. Ahmed, Gagori Bandopadhyay, Beth Coyle, Anna Grabowska

**Affiliations:** 1 Division of Cancer and Stem Cells, Cancer Biology, University of Nottingham, Nottingham, UK; 2 Children’s Brain Tumour Research Centre, Queens Medical Centre, University of Nottingham, Nottingham, UK

**Keywords:** Hypoxia, Cisplatin, Temozolomide, Chemoresistance, CD133, HIFs

## Abstract

**Purpose:**

Glioblastoma (GBM) is the commonest brain tumour in adults. A sub-population of cells within these tumours, known as cancer stem cells (CSCs), is thought to mediate their chemo-/radiotherapy resistance. CD133 is a cell surface marker that is used to identify and isolate GBM CSCs. However, its functional significance, as well as the relevant microenvironment in which to study CD133, have so far remained unknown. Here, we examined the effect of hypoxia on the expression of CD133 and on that of the hypoxia-related factors HIF-1α and HIF-2α, and the potential functional significance of CD133 expression on the acquisition of chemo-resistance by GBM cells.

**Methods:**

CD133, HIF-1α, HIF-2α, VEFG and (control) HPRT mRNA expression analyses were carried out on GBM cells (U251, U87 and SNB19; 2D or 3D cultures) under both normoxic and hypoxic conditions, using qRT-PCR. siRNA was used to downregulate CD133, HIF-1α and HIF-2α expression in the GBM cells, which was confirmed by flow cytometry and qRT-PCR, respectively. Drug sensitivity-related IC50 values were established using an Alamar Blue cell viability assay in conjunction with the Graphpad prism software tool.

**Results:**

We found that the expression of CD133 was upregulated under hypoxic conditions in both the 2D and 3D GBM cell culture models. In addition, an increased resistance to cisplatin, temozolomide and etoposide was observed in the GBM cells cultured under hypoxic conditions compared to normoxic conditions. siRNA-mediated knockdown of either HIF-1α or HIF-2α resulted in a reduced CD133 expression, with HIF-2α having a more long-term effect. We also found that HIF-2α downregulation sensitized the GBM cells to cisplatin to a greater extent than HIF-1α, whereas CD133 knockdown had a more marked effect on cisplatin sensitisation than knockdown of either one of the HIFs, suggesting the existence of a HIF-independent cisplatin resistance mechanism mediated by CD133. This same mechanism does not seem to be involved in temozolomide resistance, since we found that HIF-1α downregulation, but not HIF-2α or CD133 downregulation, sensitized GBM cells to temozolomide.

**Conclusions:**

From our data we conclude that the mechanisms underlying hypoxia-induced CD133-mediated cisplatin resistance may be instrumental for the design of new GBM treatment strategies.

**Electronic supplementary material:**

The online version of this article (10.1007/s13402-018-0374-8) contains supplementary material, which is available to authorized users.

## Introduction

Glioblastoma (GBM) is a World Health Organization (WHO) defined grade IV astrocytoma, which is the most aggressive form of glioma [1]. Despite notable achievements that have been made in the past, the clinical management of GBM patients is still a major challenge [2], which may primarily be attributed to its heterogeneous nature [3]. Temozolomide is an oral alkylating agent that is able to traverse the blood-brain barrier but, although promising results have been obtained in the standard care for GBM [4–6]**,** its efficacy ultimately diminishes due to the aquisation of resistance [7]. Cisplatin has been used as neoadjuvant therapy with temozolomide [8] and as adjuvant therapy with carmustine in patients with malignant gliomas [9]. In heavily pre-treated patients with relapsed high-grade gliomas refractory to temozolomide alone, combinations of temozolomide and cisplatin have been moderately effective [10]. When used as a first-line chemotherapy with fractionated temozolomide in chemotherapy-naïve patients with recurrent GBM, it was found to result in a progression-free survival (PFS) of 6 months [11]. Resistance to cisplatin does, however, arise, potentially due to high levels of glutathione-S-transferase (GST) activity [12]. Etoposide has been found to improve the survival of patients with high-grade glioma [13], but finally most patients succumb to GBM as a result of relapse.

The tumour microenvironment consists of a myriad of factors, including extracellular cellular matrix (ECM) components [14] and variations in oxygen tension [15], that may affect tumour biology. It has also been shown that the ECM composition may affect cancer cell proliferation and motility [16]. GBMs are characterized by regions of hypoxia [17] ranging from 0.1% to 10% [18] which are thought to play important roles in the aggressiveness of the tumours [18]. As such, hypoxia has been considered to serve as a biomarker for a poor prognosis, and to be associated with radio-resistance and chemo-resistance, as well as tumour cell migration and invasion [19]. Hypoxic regions have also been shown to harbour GBM stem cell populations [20]. Hypoxia inducible factors (HIFs) have been postulated to be the primary mediators of hypoxic responses [21]. The HIF factors HIF-1α and HIF-2α are known to be able to bind to the same DNA sequence, referred to as hypoxia responsive element (HRE), and to heterodimerise with HIF-1β to activate target genes. However, HIF-1α and HIF-2α may also exhibit functional differences, including different protein-protein interactions [22] and tissue specificities [23]. HIF-1α has been found to promote the survival of glioma cancer stem cells under hypoxic conditions [24], and HIF-2α is thought to be linked to the GBM stem cell phenotype within the hypoxic niche [20]. Additionally, it has been found that in neuroblastoma cells exposed to hypoxia, the expression of HIF-1α rapidly peaks and declines, whereas that of HIF-2α remains steadily upregulated, thereby mediating a long lasting response [23].

Since cancer stem cells (CSCs) have been implicated in tumour recurrence and drug failure, their identification and characterization is of therapeutic relevance [25, 26]. Several markers have been employed to differentiate CSCs from the vast majority of cells within a tumour sample [27]. In GBM, CD133 is commonly used as a CSC marker [28]. As yet, however, only a few studies have been performed on the biological significance of CD133 in GBM. In addition, the most appropriate environment in which to study GBM cells has been contentious. Most assays used so far do not take into account the influence of the tumour microenvironment (TME) and its role in regulating CSC numbers and phenotypes. Tumour cells, including GBM cells, are traditionally cultured in vitro with an oxygen tension of 20%. Since this may not accurately reflect the behaviour of tumours in the in vivo situation, it may explain why 95% of the anti-cancer drugs tested in vitro have failed their translation to the clinic [29].

In the present study, we sought to understand the role of hypoxia on CD133 expression, and whether or not HIFs play any regulatory role in this expression. We also assessed the role of hypoxia in etoposide and cisplatin chemo-resistance and how HIF and CD133 downregulation may sensitize GBM cells to chemotherapy. Our results strongly indicate that CD133 upregulation under hypoxic conditions is mediated by HIFs, with a longer-lasting effect of HIF-2α. In addition, we found that CD133 knockdown sensitized GBM cells to cisplatin in a HIF-independent manner.

## Materials and methods

### Cell lines and culture conditions

U87 cells were derived from the European Collection of Authenticated Cell Cultures (ECACC), while U251 and SNB19 cells were derived from the National Cancer Institute USA (NCI-60). The GBM cells were grown under normoxic (20% oxygen) or hypoxic (1% oxygen) conditions as standard 2D cultures or as Cultrex-based 3D cultures (Trevigen, Gaithersburg, MD, USA).

### Quantitative real-time PCR

RNA extraction, cDNA synthesis and quantitative real-time RT-PCR (qRT-PCR) were performed as previously reported [30] using SYBR green (Eurogentec) for detection. The data were expressed relative to the housekeeping gene *HPRT* and calculated using the 2^-∆∆Ct^ method. The primer sequences used were: HPRT (F) 5’-ATTATGCTGAGGATTTGGAAAGGG-3′ and (R) 5’-GCCTCCCATCTCCTTCATCAC-3′; CD133 (F) 5’-CAATCTCCCTGTTGGTGATTTG-3′ and (R) 5’-ATCACCAGGTAAGAACCCGGA-3′; VEGF (F) 5’-CCAAGTGGTCCCAGGCTGCA-3′ and (R) 5’-TGGATGGCAGTAGCTGCGCT-3′; HIF1A (F) 5’-CCTCTGTGATGAGGCTTACCATC-3′ and (R) 5’-CATCTGTGCTTTCATGTCATCTTC-3′, HIF2A (F) 5’-CCACCAGCTTCACTCTCTCC-3′ and (R) 5’-TCAGAAAAGGCCACTGCTT-3′.

### Small interfering RNA transfections

GBM cells were transfected with CD133, HIF-1α and HIF-2α siRNAs (Eurogentec) using a Lipofectamine® RNAiMAX Transfection Reagent (Life Technologies) according to manufacturer’s instructions. The sequences used were: CD133siRNA- GAUCAAAAGGAGUCGGAAA, HIFIAsiRNA- GCCACUUCGAAGUAGUGCU and HIF2AsiRNA- GCGACAGCUGGAGUAUGAA.

### 3D cultures

Cultrex basement membrane extract (BME; Trevigen) was diluted to a concentration of 3 mg/ml on ice using phenol red-free modified RPMI-1640 medium (Life Technologies). Next, the cells were resuspended at appropriate densities and seeded into black-walled, low-adherent, clear-bottom 96-well culture plates (BrandTech) prewarmed to 37 °C.

### Drug sensitivity assays

A cisplatin stock solution of 1 mg/ml was diluted to appropriate concentrations. GBM cells were subsequently incubated with drugs for 48 h after which an Alamar Blue cell viability assay (Invitrogen) was carried out (10% v/v, 37 °C, 1 h). The resulting fluorescence was measured using a fluorescence plate reader (Flex-Station II, Molecular Devices, CA, USA) and IC50 values were calculated relative to untreated cells using the Graphpad prism software tool. Drug sensitivities were calculated as percentages of matched untreated controls. IC50 curves were plotted and values determined using GraphPad Prism 6 (GraphPad Software Inc., USA; nonlinear curve fit of *Y* = 100/ (1 + 10^(LogIC50-X)*^HillSlope).

### Flow cytometry

After harvesting, GBM cells were spun down in Eppendorf tubes and re-suspended in 80 μl buffer and 20 μl FcR blocker (Miltenyi Biotec), after which 10 μl anti-CD133/1 (AC133)-PE antibody (Miltenyi Biotec) or a mouse monoclonal IgG-PE isotype control was added according to the manufacturer’s instructions. Next, the cells were analysed using a Beckman Coulter flow cytometer. The resulting data were analysed using Weasel software (http://www.frankbattye.com.au/Weasel/).

### Statistic analyses

Graphpad Prism version 6 was used to analyse all data. Data comparisons were carried out using either Student’s t-test or one-way ANOVA (Turkey’s multiple comparison test), when appropriate.

## Results

### CD133 expression is increased over time following exposure to hypoxia

**Fig. 1:**
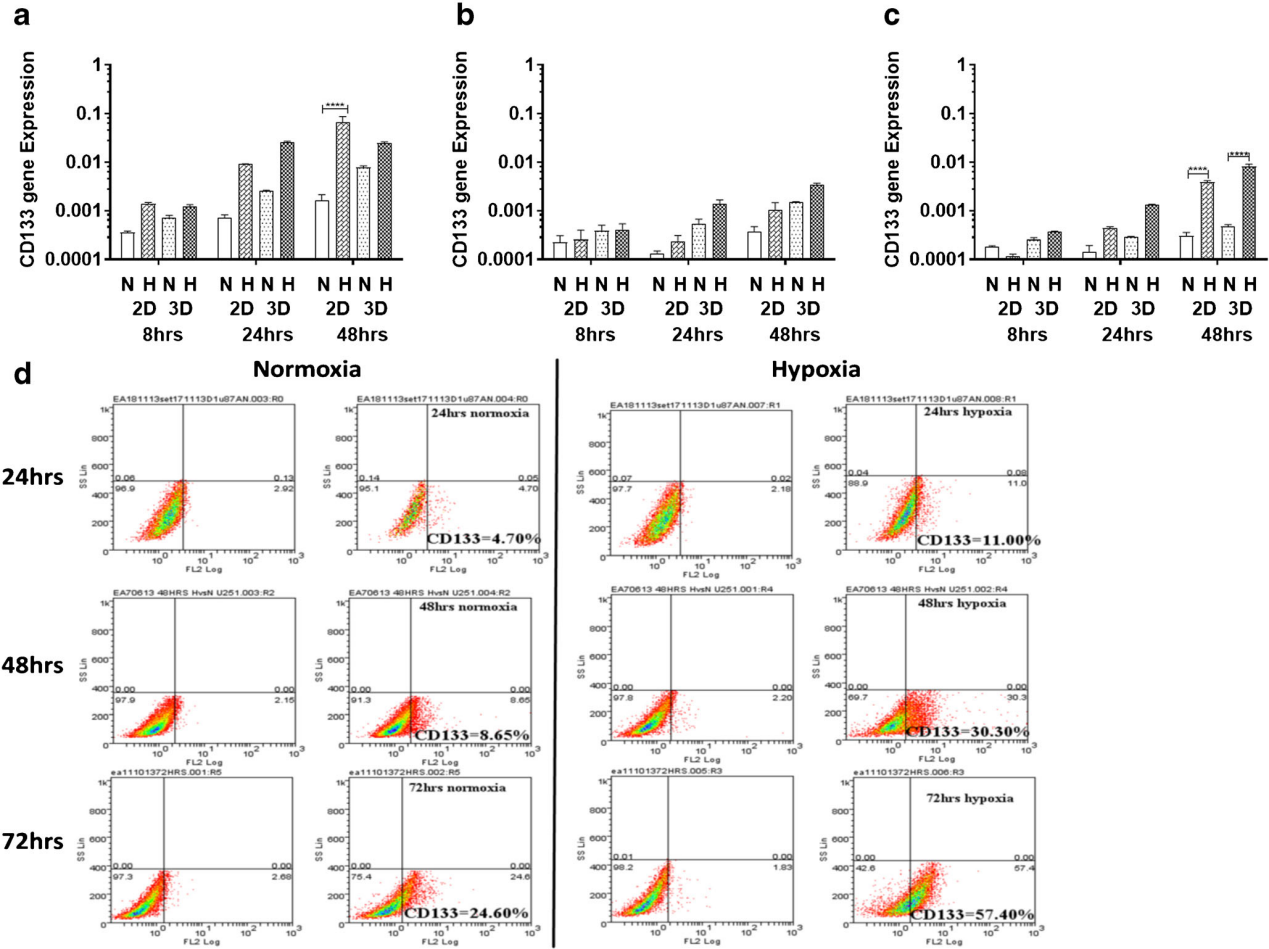
**CD133 is upregulated under hypoxic conditions**. U251 (**a**), U87 (**b**) and SNB19 (**c**) cells were cultured as 2D and 3D models under normoxic (N) or hypoxic (H) conditions. CD133 mRNA levels were quantified using qRT-PCR at the indicated times. The error bars represent standard errors of means of the average of 3 independent experiments with triple replicates per experiment for U251 and U87 and one experiment for SNB19. One way ANOVA (Prism6) was used for statistical comparison. ****p ˂ 0.0001 (**d**) Flow cytometric analysis of CD133 in U251 cells cultured in 2D in a 96-well plate at a density of 10,000 cells/well. The cells were divided into two sets: normoxia (left) and hypoxia (right). For both sets, the total isotype control cell populations are presented based on side and scatter properties, and appropriate regions are gated and used to compare cells stained with the anti-CD133 antibody. The percentages of cells expressing CD133 after 24 to 72 h are indicated. The analyses were performed using Weasel software

**Fig. 2:**
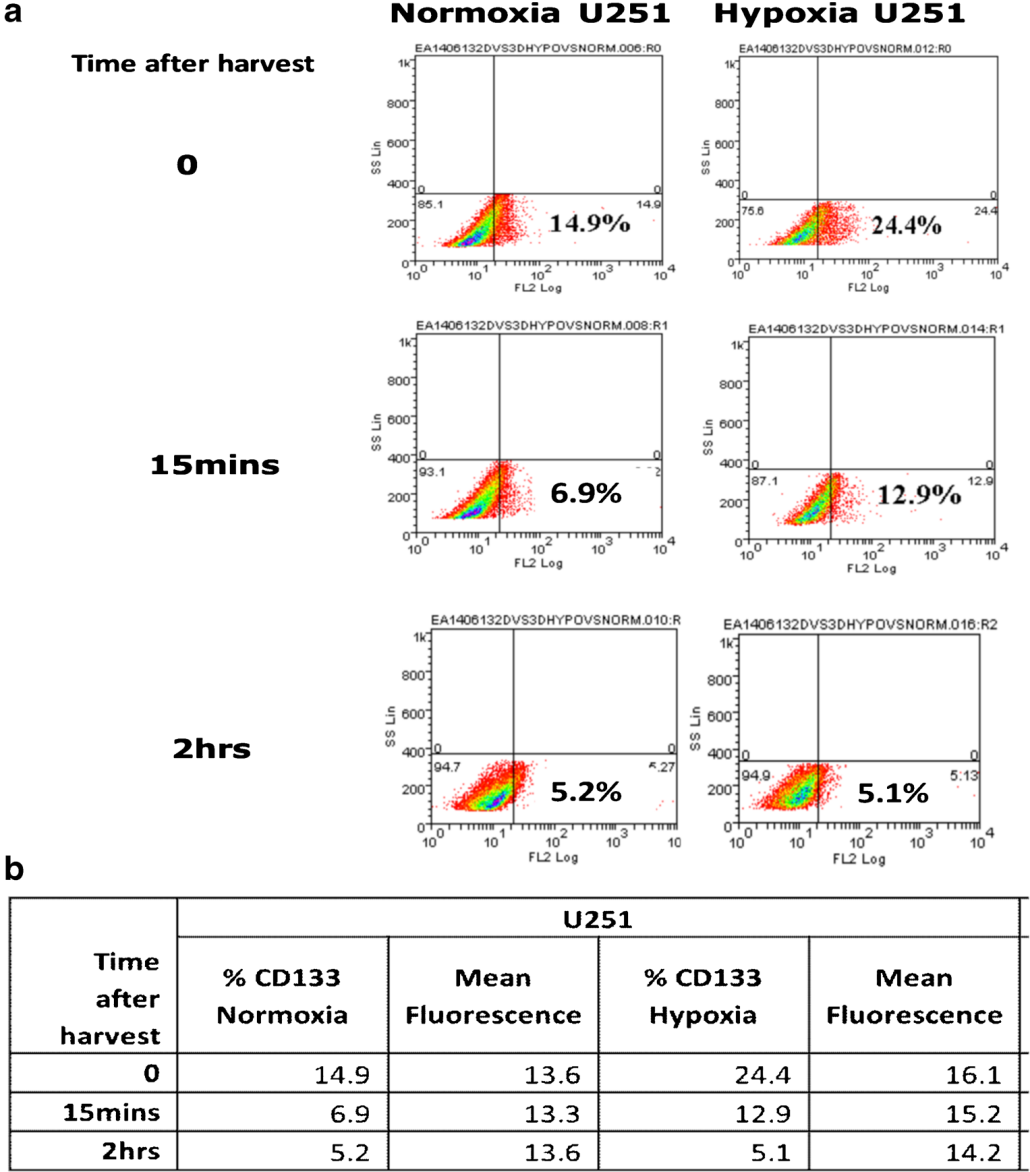
**CD133 protein expression in U251 cells over time**. **a** Expression of CD133 in U251 cells cultured in 2D under normoxic (left column) and hypoxic (right column) conditions. In the top row (0) the cells were stained immediately after harvesting with EDTA. In the middle row the cells were stained 15 mins after harvesting. In the bottom row the cells were stained 2 hrs after harvesting. The percentages of cells expressing CD133 overtime are indicated. **b** CD133 expression in U251 cells over time and mean florescence scores. The analyses were performed using Weasel software

To determine the effect of hypoxia on the expression of CD133, we examined three glioblastoma (GBM)-derived cell lines, U251, U87 and SNB19, cultured either in 2D or 3D under normoxic (20% oxygen) or hypoxic (1% oxygen) conditions. We found that all three cell lines were viable in both culture systems grown under both normoxic and hypoxic conditions (Supplemental Fig. 1). We also found that CD133 expression was higher under hypoxic compared to normoxic conditions (Fig. 1a–c), with a significant upregulation in the U251 and SNB19 2D models and the SNB19 3D model after 48 h, respectively, using qRT-PCR. In parallel, VEGF mRNA expression was found to be induced (Supplemental Table 1A–C). This result is indicative of an important role of the GBM microenvironment in CD133 expression. An increase in hypoxia-induced CD133 expression over time was subsequently confirmed at the protein level using flow cytometry in the 2D models (Fig. 1d, Supplemental Fig. 2). CD133 protein expression in the 3D cultures turned out to be technically challenging due to the time required to harvest cells from the 3D matrix. We found that in U251 cells cultured in 2D under normoxic or hypoxic conditions CD133 expression was rapidly lost (0 h, 15 min and 2 h measurements). For cells grown in normoxia the CD133 protein expression levels were found to be 14.9%, 6.9% and 4.4%, and that for cells grown in hypoxia 24.4%, 12.9% and 5.1%, respectively (Fig. 2a, b).

### HIFs regulate CD133 expression in a time-dependent manner

**Fig. 3:**
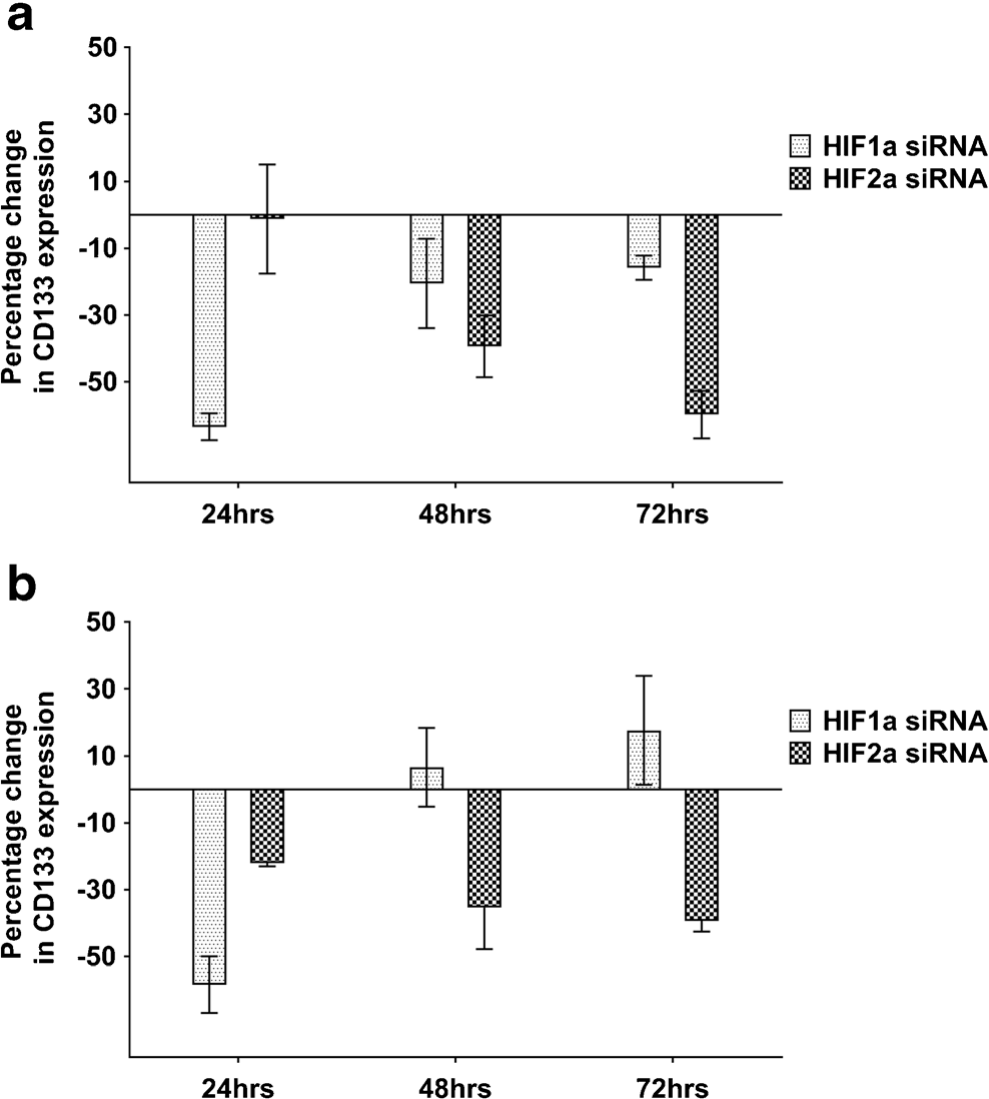
**HIF-1α and HIF-2α regulate CD133 expression in a time-dependent manner**. U251 cells were transfected with siRNAs specific for HIF-1α or HIF-1α and subjected to hypoxia (1% oxygen) within 1 h of transfection. At day 1 after transfection the cells were seeded into 96-well plates at a density of 10,000 cells/well in 2D (**a**) or 3D (**b**). CD133 mRNA expression levels were monitored over time relative to non-targeting siRNA controls. Error bars represent standard errors of the means from 2 independent experiments in 2D and 3 independent experiments in 3D

Since CD133 was found to be upregulated under hypoxic conditions in both 2D and 3D GBM cell models, we next set out to assess the mechanism by which CD133 is upregulated. HIF-1α and HIF-2α are known to play important roles in tumour progression under hypoxic conditions [21], but they have not been studied exhaustively with respect to CD133 expression. After we found that through siRNA transfection >57% HIF-1α and HIF-2α mRNA expression knockdown was achieved in both the 2D and 3D models (Supplemental Fig. 3A-C), we set out to assess CD133 expression in GBM cells under hypoxic conditions. In the U251 2D model following 24 h exposure to hypoxia, which corresponds to a mild/acute hypoxia exposure, we found that while HIF-1α knockdown led to 63% CD133 expression downregulation, little effect was noted after HIF-2α knockdown (Fig. 3a). However, at later timepoints, corresponding to a chronic/prolonged hypoxia exposure, we found that CD133 expression in the HIF-1α silenced cells started to return to baseline, while in the HIF-2α silenced cells CD133 expression downregulation increased to 39% at 48 h and 69% at 72 h, respectively (Fig. 3a). Similar results were obtained with U251 (Fig. 3b) and U87 cells (Supplemental Fig. 3D) cultured in 3D. In parallel, we again observed VEGF expression induction after HIF-1α or HIF-2α knockdown in the 3D model (Supplemental Fig. 3E, F), suggesting a gradual shift from a HIF-1α dependent to a HIF-2α dependent effect after chronic hypoxia exposure.

### GBM cells are resistant to chemotherapy under hypoxic conditions

**Fig. 4:**
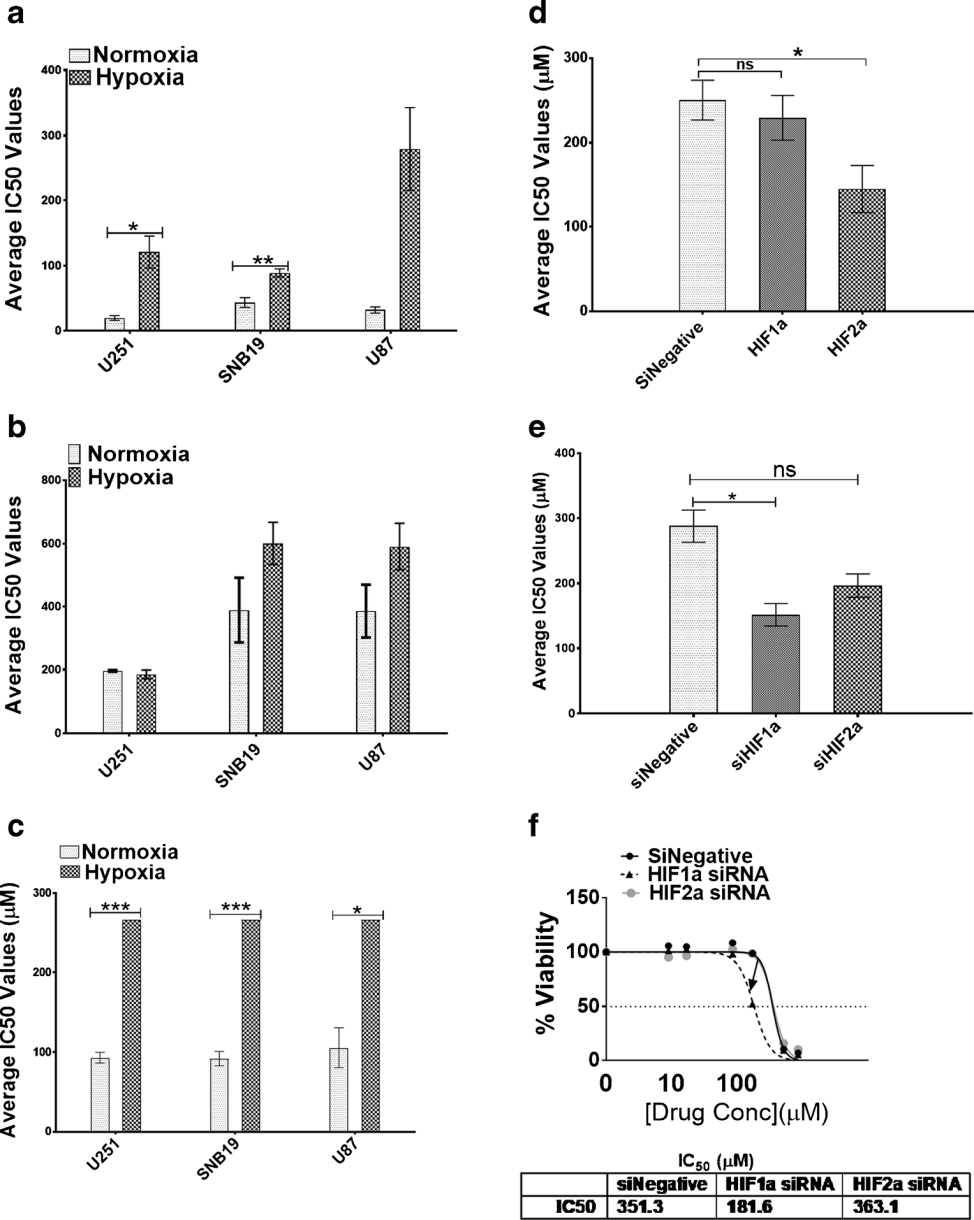
**GBM cells are resistant to chemotherapy when cultured under hypoxic conditions.** Mean IC_50_ values (μM) for GBM cells under normoxic and hypoxic conditions following exposure to cisplatin (**a**), temozolomide (**b**) and etoposide (**c**) based on Alamar Blue assays. Error bars represent standard errors of the means from 3 independent experiments.*p < 0.05, **p < 0.01,***p < 0.001 (Student t-test). **d** After HIF-1α and HIF-2α downregulation in U251 cells, the mean IC_50_ values (μM) of cisplatin were evaluated based on Alamar Blue assays. Error bars represent standard errors of the means from 3 independent experiments.*p < 0.05 (Student t-test). **e** After HIF-1α and HIF-2α downregulation, U251 cells were treated with temozolomide after which cell viabilities were ascertained using Alamar Blue assays. Error bars represent standard errors of means from 3 independent experiments.*p < 0.05, ns = not significant. (**f**) Pattern of IC_50_ shift to the left in U87 cells following HIF-1α but not HIF-2α downregulation. IC_50_ values for etoposide were not achieved. Values indicate the highest concentration used

We next explored the effect of hypoxia on the cisplatin, temozolomide and etoposide chemo-sensitivities of GBM cells. We observed an increase in cisplatin resistance in the three cell lines following exposure to hypoxia compared to those cultured under normoxic conditions (Fig. 4a, Supplemental Table 2A), but this increase was only statistically significant in U251 and SNB19 cells. In addition, we observed increases in temozolomide resistance in the U87 and SNB19 cells, but these increases were not statistically significant (Fig. 4b). No IC50 values could be obtained for etoposide in the GBM cells under hypoxic conditions, even at the highest concentration (267 μM) used (Fig. 4c, Supplemental Table 2B). Therefore, we conclude that all three GBM cell lines are >2-fold resistant to etoposide under hypoxic conditions compared to normoxic conditions (Fig. 4c, Supplemental Table 2B).

As HIFs are known to act as key mediators of hypoxia (see above), we next investigated the effects of HIF-1α and HIF-2α downregulation on GBM drug sensitivity. Since we found that IC50 values for etoposide under hypoxia could not be obtained, only sensitivities to cisplatin and temozolomide were investigated. In doing so, we found that HIF-2α downregulation, but not HIF-1α downregulation, significantly sensitized U251 and U87 cells to cisplatin (Fig. 4d, Supplemental Fig. 4, Supplemental Table 2C). In contrast, we found that HIF-1α downregulation, but not HIF-2α downregulation, significantly sensitized these GBM cells to temozolomide, i.e., > 47% and 48% in U251 and U87, respectively (Fig. 4e, f).

### CD133 downregulation sensitizes GBM cells to cisplatin, but not temozolomide

**Fig. 5:**
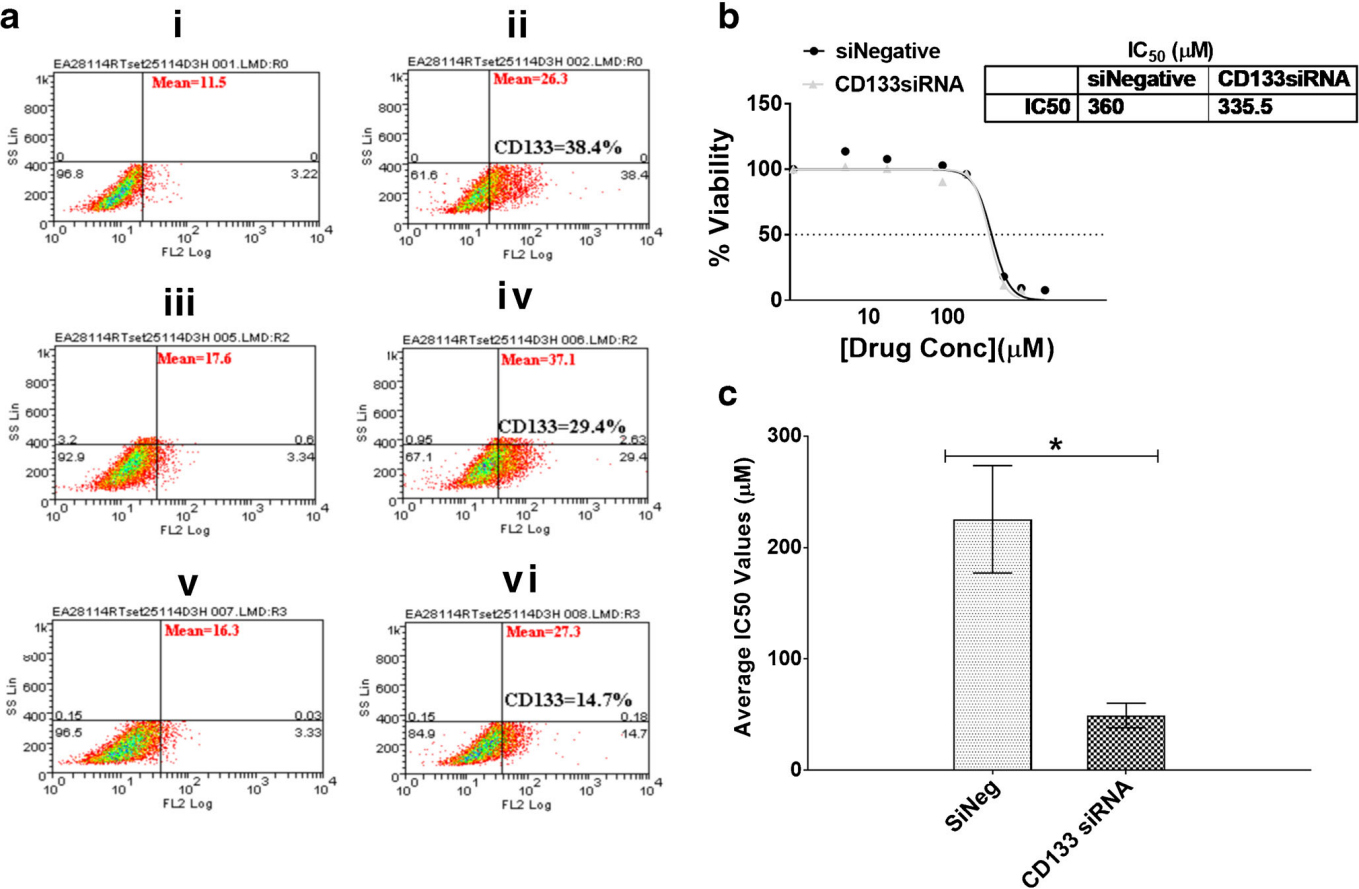
**CD133 downregulation sensitizes glioblastoma cells to cisplatin**. **a** U251 cells were seeded in 6-well plates at a density of 500,000 cells/well. At day 0 the cells were transfected with either a non-targeting siRNA (middle panel) or a CD133 specific siRNA (bottom panel). Untransfected cells were also included as control (upper panel). The cells were subjected to hypoxia at day 0 of transfection. At day 3 post transfection, the cells were harvested and stained with an anti-CD133 antibody. The total population in the isotype control cells are presented based on scatter properties in the dot plot (i, iii and v), and appropriate regions were gated for the three sets of cells used to compare CD133 positive cells (ii, iv, and vi). CD133 expression levels as analysed by Weasel software were 38.4% (ii), 29.8% (iv) and 14.9% (vi). The mean fluorescence intensities of the positive cells are as presented in the plots. A mouse monoclonal IgG was used as isotype control. **b** Following CD133 downregulation, U251 cells were treated with temozolomide and their viability ascertained using Alamar Blue assays. **c** Following CD133 downregulation, U251 cells were treated with cisplatin and their viability ascertained using Alamar Blue assays. Error bars represent standard errors of means from 3 independent experiments.*p < 0.05

Although HIF-1α and HIF-2α were found to be involved in the regulation of CD133 expression under hypoxia, we noted that their knockdown did not completely ablate CD133 expression, suggesting that its encoding gene may be regulated by yet another mechanism(s). In addition we found that, while HIF-1α affected the temozolomide sensitivity of GBM cells, HIF-2α knockdown affected their cisplatin sensitivity. Therefore, we next set out to investigate the possibility that CD133, independently of HIFs, may be involved in temozolomide and cisplatin resistance. Following CD133 expression knockdown, we observed a 2-fold decrease in CD133 protein expression as assessed by flow cytometry (Fig. 5a). This decrease did not result in sensitization of the different GBM cells to temozolomide (Fig. 5b, Supplemental Fig. 5A). Interestingly, however, we observed a significant 5-fold increase in sensitivity to cisplatin in U251 cells (Fig. 5c, Supplemental Table 3) and 2-fold increases in this sensitivity in U87 and SNB19 cells (Supplemental Fig. 5B, C; Supplemental Table 3). These results indicate that CD133 silencing enhances the efficacy of cisplatin in the treatment of GBM.

## Discussion

The emerging concept that tumour development largely relies on signals derived from the tumour microenvironment (TME) has led to a paradigm shift in cancer research [31]. Locally advanced tumours are frequently characterized by hypoxia resulting from abnormal micro-vessel structure and function, and increased distances between blood vessels and tumour cells [32]. Hypoxia is an important feature of the glioblastoma (GBM) TME [20] and cells within their hypoxic niches have been shown to be chemo-resistant [33]. Here, we observed CD133 expression upregulation in GBM cells cultured in 2D and 3D models under hypoxic conditions and, although largely consistent with other reports, different CD133 levels were observed in the different GBM cells tested [25, 34]. CD133 expression upregulation under hypoxic conditions has been reported before [35]. Our current data indicate that hypoxia acts on GBM cells irrespective of whether they are cultured in 2D or 3D. Although no significant CD133 upregulation was reached in the 3D model, a consistent higher level of CD133 expression was observed when the cells were grown in 3D, similar to what has recently been reported for GBM cells grown in a 3D chitosan-alginate scaffold [36]. CD133 protein expression was also verified in the 2D models using flow cytometry. We found, however, that this expression rapidly decreased after harvesting of the cells, making it technically challenging to assess CD133 expression in the 3D cultures, which require additional manipulations to free and disaggregate the GBM cell clusters. This observation may also explain previous discrepancies noted in the analysis/sorting of CD133 positive cell populations via flow cytometry and why apparently CD133 negative cells may give rise to CD133 positive cells.

In both the 2D and 3D GBM cell models we found that HIF-1α and HIF-2α knockdown led to a reduced CD133 expression. While the effect of HIF-1α silencing on CD133 expression diminished rapidly, we found that the effect of HIF-2α silencing on CD133 expression became stronger over time, similar to its effect on VEGF expression, which is a known HIF downstream target. This finding is in keeping with a previous report on neuroblastoma cells [23]. The underlying mechanism may involve the hypoxia-associated factor HAF, which becomes upregulated during acute hypoxia and selectively binds to HIF-1α, resulting in its degradation, while at the same time HIF-2α transactivation and stability are promoted [37]. SIRT1, a histone deacetylase, also appears to be involved in this switch, i.e., it may deacetylate HIF-1α and reduce its activity while enhancing HIF-2α-mediated transcription through a HIF-dependent mechanism [38].

Chemo-resistance plays a crucial role in GBM tumour progression [39, 40] and it has been found that cells located in its hypoxic regions are more resistant to chemotherapy and may give rise to recurrences [17]. Our current results also indicate that GBM cells become more resistant to temozolomide, cisplatin and etoposide under hypoxic conditions. This, however, is not a universal finding. While in one study breast cancer cells exposed to low oxygen levels (< 0.1%) were also found to be resistant to a range of chemotherapeutic agents, others have found differential responses of cells to therapeutic drugs under hypoxic conditions, with cisplatin being more effective in hypoxic breast cancer, small cell lung cancer and lymphoma cells compared to the same cells maintained under normoxic conditions [41]. The mechanism underlying this drug resistance may be cell type dependent. In the breast cancer cells, it has been found that enhanced resistance to cisplatin under hypoxic conditions was mediated through HIF-1α [42], whereas in osteosarcoma cells hypoxia-induced resistance to cisplatin, doxorubicin and etoposide has been found to be HIF-independent and to occur via hypoxia-driven attenuation of p53 activation [43]. In the present study, we found that siRNA-mediated HIF-1α knockdown, but not HIF-2α knockdown, significantly sensitized GBM cells to temozolomide. Others have similarly found that HIF-1α downregulation by BMP2 may increase the responsiveness of GBM cells to temozolomide [44]. Our findings are also in agreement with those from Li et al. who found that HIF-1α silencing increases the sensitivity of tumour cells to temozolomide in vivo [45].

We also found that HIF-2α downregulation sensitized GBM cells to cisplatin more effectively than HIF-1α downregulation, substantiating the differential roles of HIF-1α and HIF-2α in tumour development [46]. This effect of HIF downregulation on drug sensitivity was, however, relatively small compared to the effect of CD133 downregulation, which resulted in an increase in cisplatin sensitivity of at least 2-fold in all GBM cells tested and as much as 7-fold in U251 cells. In contrast, we found that CD133 downregulation did not significantly affect the temozolomide sensitivity of GBM cells. This latter observation is in accordance with that of Perazzoli et al. who concluded from their results that CD133 was unrelated to temozolomide resistance [47], but disagrees with others that have shown that CD133^+^ GBM cells are resistant to temozolomide [48]. Our results suggest that the hypoxia-induced cisplatin sensitivity of GBM cells may be HIF independent and may be directly or indirectly induced via CD133 activation. One potential mechanism for CD133-dependent resistance may be CD133-mediated activation of the Erk pathway, which has indeed been observed after CD133 overexpression in GBM cells [49]. Interestingly, phosphorylation of the tyrosine-828 residue in the C-terminal cytoplasmic domain of CD133 has been found to mediate direct interactions between CD133 and the phosphoinositide 3-kinase (PI3K) 85 kDa regulatory subunit (p85), resulting in preferential activation of the PI3K/protein kinase B (Akt) pathway in GBM stem cells compared to matched non-stem cells [50]. Therefore, upregulation of CD133 in GBM cells under hypoxic conditions may lead to activation of the anti-apoptotic Akt pathway, resulting in drug resistance [51].

Although cisplatin as a neoadjuvant agent, alone or in combination with other agents including temozolomide, has been explored and some benefits have been noted [8–11], eventually resistance may occur resulting in a poor clinical outcome. While HIF-dependent and HIF-independent mechanisms of cisplatin resistance have previously been described, we identified a novel mechanism of hypoxia-induced cisplatin resistance that is mediated via CD133. This mechanism may also, at least partly, explain the observed association between CD133 expression and a poor prognosis [52], and suggests a role for CD133 as a functional molecule in its own right rather than as a CSC marker. A further in-depth investigation of the mechanism underlying this hypoxia-induced CD133-dependent cisplatin resistance may facilitate the identification of cisplatin combination therapies to improve its efficacy for clinical use in neo-adjuvant and/or adjuvant settings.

## Electronic supplementary material

ESM 1 (DOCX 1775 kb)

## Abbreviations

2D: 2-Dimensional
3D: 3-Dimensional
CD133: Cluster of differentiation 133
CSC: Cancer stem cells
FACS: Fluorescence activated cell sorting
GBM: Glioblastoma multiforme
HIF1a: Hypoxia inducible factor 1
HIF2a: Hypoxia inducible factor 2
HPRT: Hypoxanthine phosphoribosyltransferase
mRNA: Messenger RNA
RNA: Ribonucleic acid
qRT-PCR: Quantitative real-time polymerase chain reaction
siNeg: Small interfering RNA negative
siRNA: Small interfering RNA
TME: Tumour microenvironment
TMZ: Temozolomide
VEGF: Vascular endothelial growth factor
WHO: World Health Organization
