# Supplementary material for: A HIF-independent, CD133-mediated mechanism of cisplatin resistance in glioblastoma cells
Source: Cell Oncol (Dordr). 2018 Feb 28;41(3):319–28. doi: 10.1007/s13402-018-0374-8 (PMC5951876; doi:10.1007/s13402-018-0374-8)
Supplement: Supplementary file 1 — (DOCX 1775 kb) [file 13402_2018_374_MOESM1_ESM.docx]

**TABLES**

**
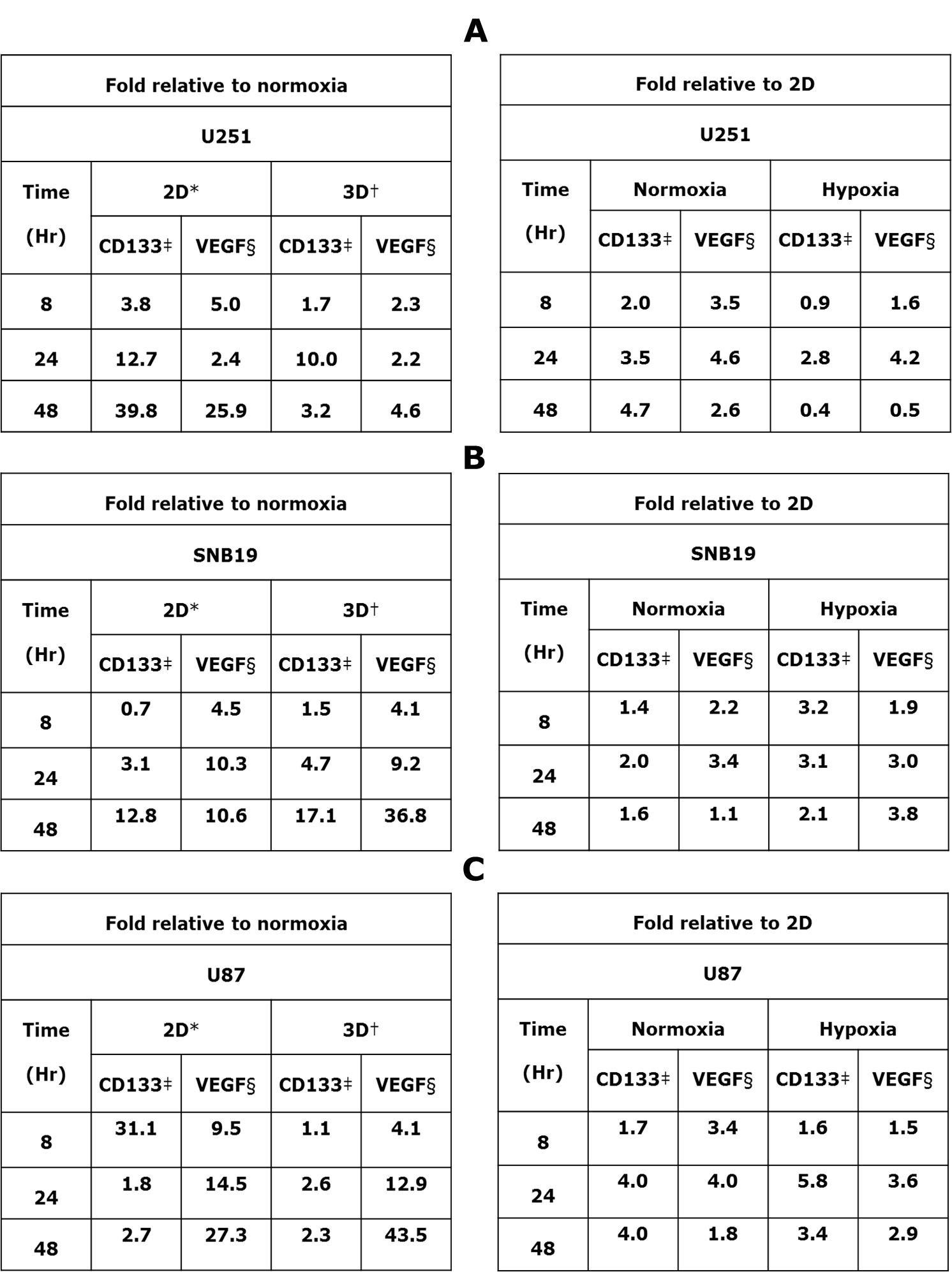
**

**Supp. Table 1. Fold difference and level of CD133 mRNA expression relative to normoxia and 2D**. U251 (A), U87 (B), and SNB19 (C) glioblastoma cell lines were cultured in the 2D and 3D models. The expression of CD133 was quantified at the indicated times with qRT-PCR and the average fold difference relative to normoxia and 2D was calculated (n=3 for U251 and U87; n=1 for SNB19.

*2-Dimensional, †3-Dimensional, ‡Cluster of Differentiation 133, §Vascular endothelial growth factor


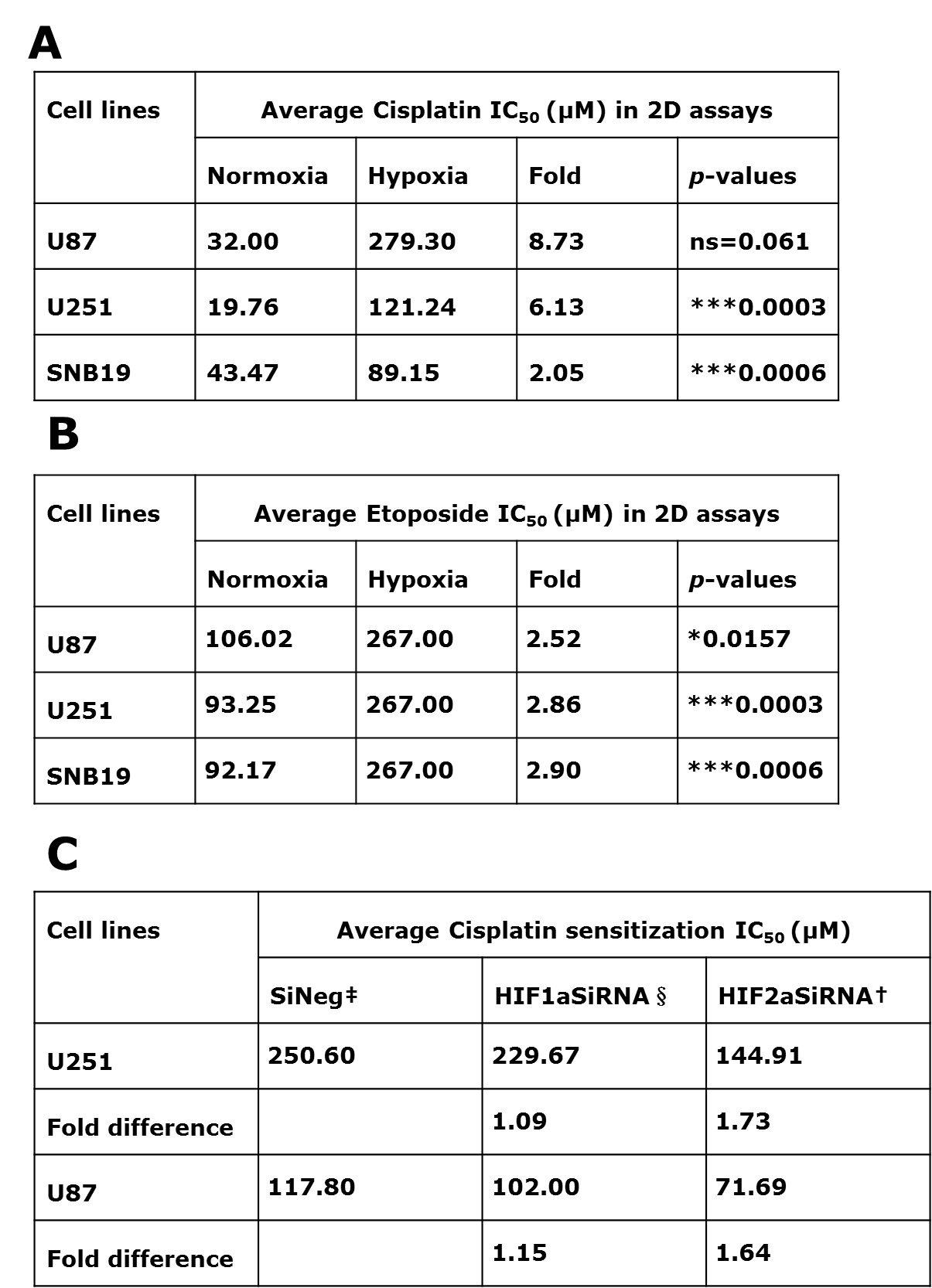


**Supp table 2: Table showing average IC_50_ values.** (A and B) fold difference in cisplatin (A) and etoposide (B) following exposure to hypoxia. N=3. (C) Following downregulation of HIF1a and HIF2a, the fold-difference in cisplatin sensitization was evaluated in U251 and U87 cell lines. N=3 for U521 and 1 for U87**.** NS=not significant. **p*<0.05, ***p*<0.01, ****p*<0.001

‡ Small interfering RNA negative, §Hypoxia Inducible Factor 1 small interfering RNA, †Hypoxia Inducible Factor 2 small interfering RNA

**
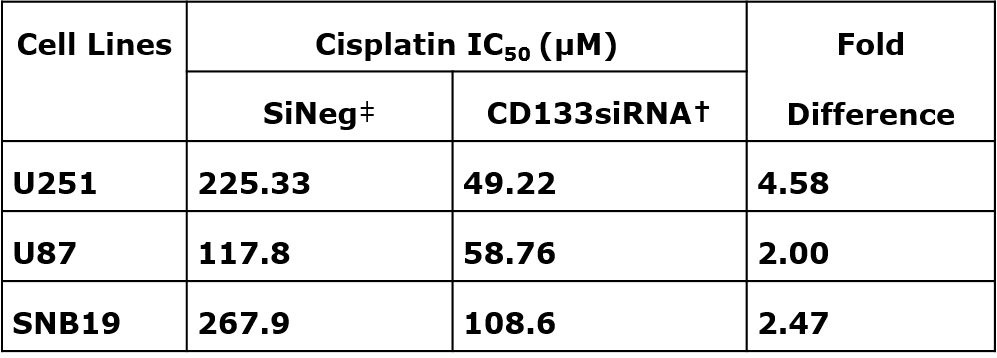
**

**Supp. Table 3 Effect of CD133 knock-down on cisplatin:** Fold difference in cisplatin sensitization following downregulation of CD133 in U251, U87 and SNB19 cells. N=3 for U251 and 1 for U87 and SNB19**.**

‡Small interfering RNA negative, **†**=Cluster of Differentiation 133 small interfering RNA


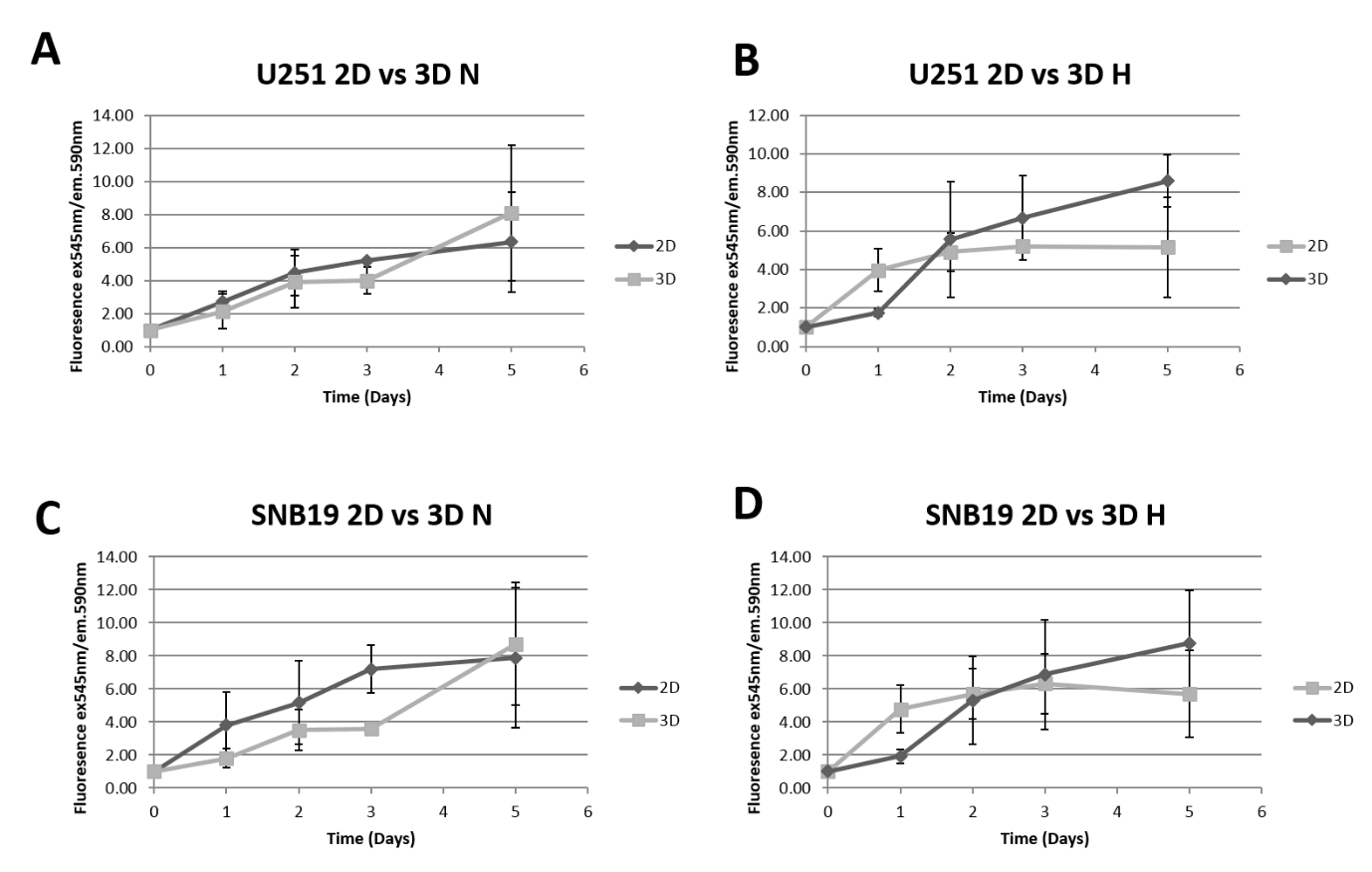


**Supp Fig. 1. Glioblastoma cells are viable in 2D and 3D models when grown in normoxia or exposed to hypoxia.** U251 (A and B) and SNB19 cells (C and D) were cultured in the 2D and 3D models. At day 0 of set up, baseline reading was taken with the AlamarBlue assay after the cells had settled and one set of the cells was maintained in normoxia (left panel) while the other set was transferred to hypoxia (right panel). The viability of the cells was monitored for 5 days. The error bars represent the SD from three independent experiments. The graph was plotted relative to day 0.


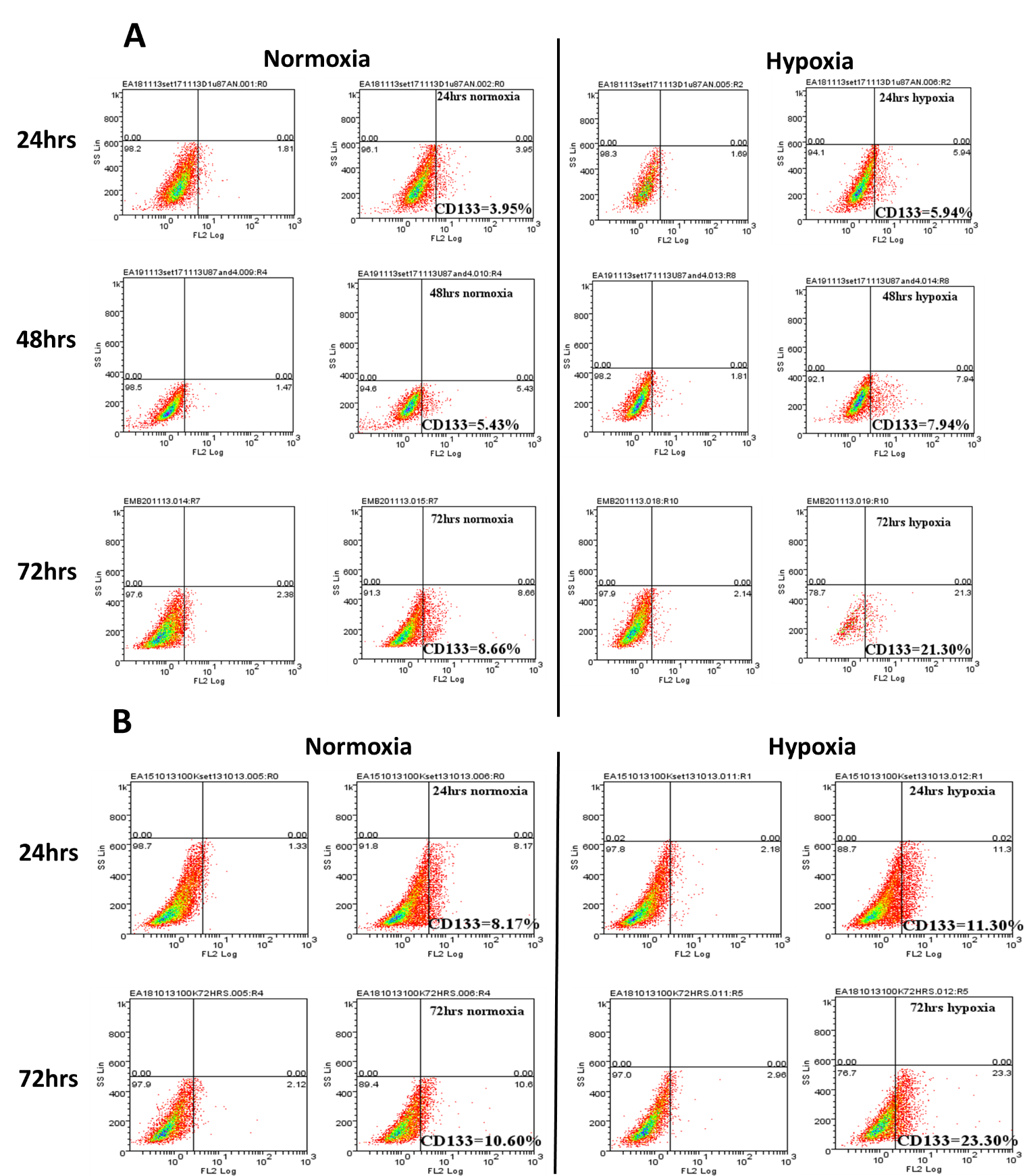


**Supp Fig. 2.** **Flow cytometric analysis of CD133 in glioblastoma cell lines:** U87 (A) and SNB19 (B) cells were cultured in a 24 well plate with 20,000 cells/well. The cells were divided into two sets-normoxia (left) and hypoxia (right). For both sets, the total population in the isotype control cells were presented based on side and scatter properties and appropriate region gated and used to compare cells stained with anti-CD133 antibody. The percentage of cells expressing CD133 overtime is as indicated. The analysis was performed with weasel software.


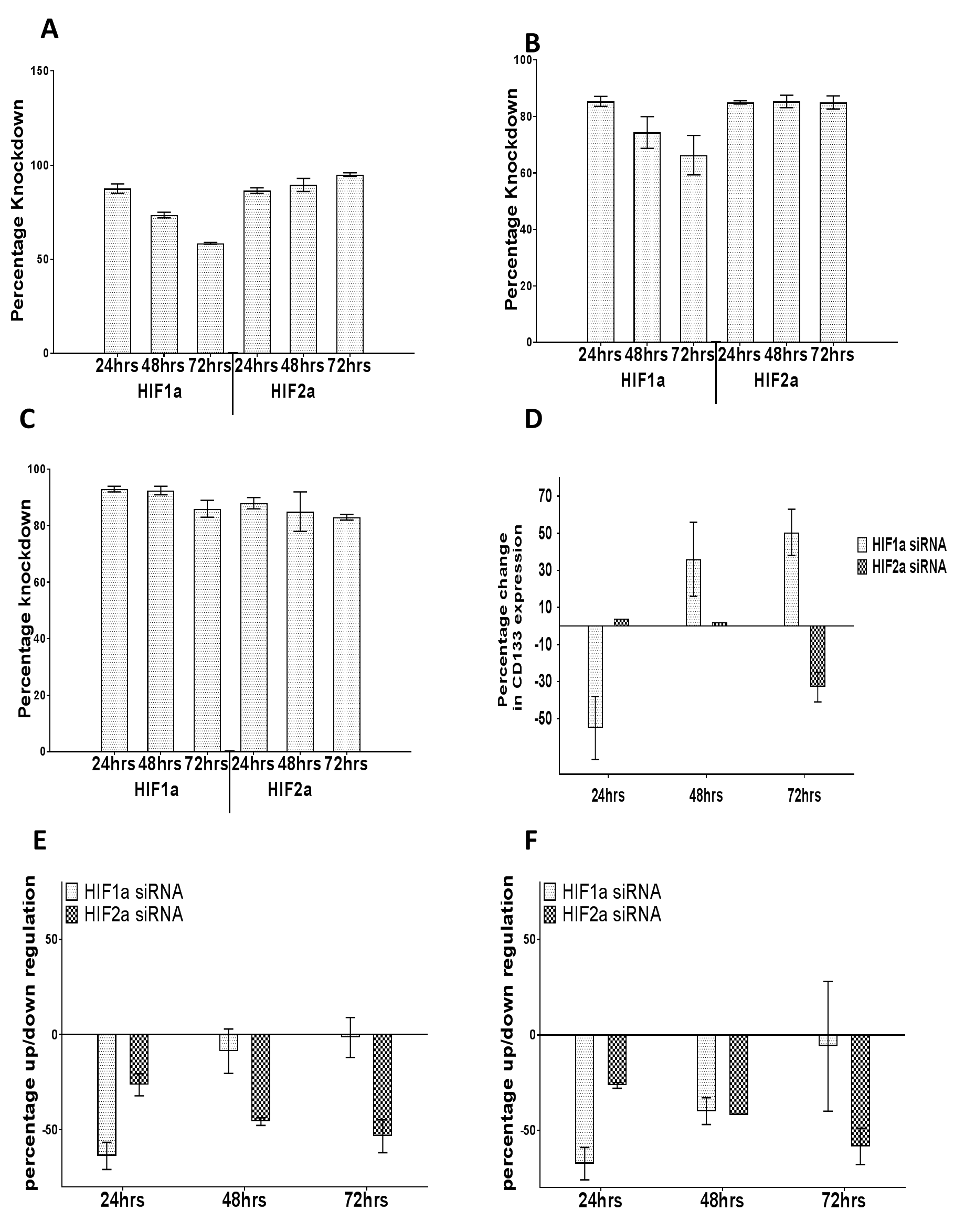


Supp Fig. 3. HIF1a and HIF2a regulate CD133 and VEGF expression in a time-dependent manner. U251 cells cultured in 2D (A) and 3D (B) models and U87 cells (C) cultured in 3D models were reverse-transfected in a 6-well plate with siRNA specific for HIF1a or HIF2a. Non-targeting siRNA was used as negative control (siNegative). The cells were transferred to hypoxia (1% oxygen) within 1hr of transfection. At day 1 of transfection, the cells were harvested and seeded at 10,000 cells/well into a 96-well plate in the respective models and HIF1a (left part of graph) and HIF2a (right part of graph) knockdown was monitored for 72hrs. Error bars represent the standard error of mean from 3 independent experiments for U251 (3D) and 2 independent experiments for U251 (2D) and U87 (3D). (D) Following downregulation of either HIF1a siRNA or HIF2a siRNA the mRNA expression of CD133 was monitored over time in U87 cells cultured in 3D model. Error bars represent the standard error of mean from 2 independent experiments. (E and F) Following the transfection with either HIF1a siRNA or HIF2a siRNA in 3D model in U251 (E) and U87 (F) cells, the mRNA expression of VEGF was monitored over time. Error bars represent the standard error of mean from 3 independent experiments for U251 and 2 independent experiments for U87.


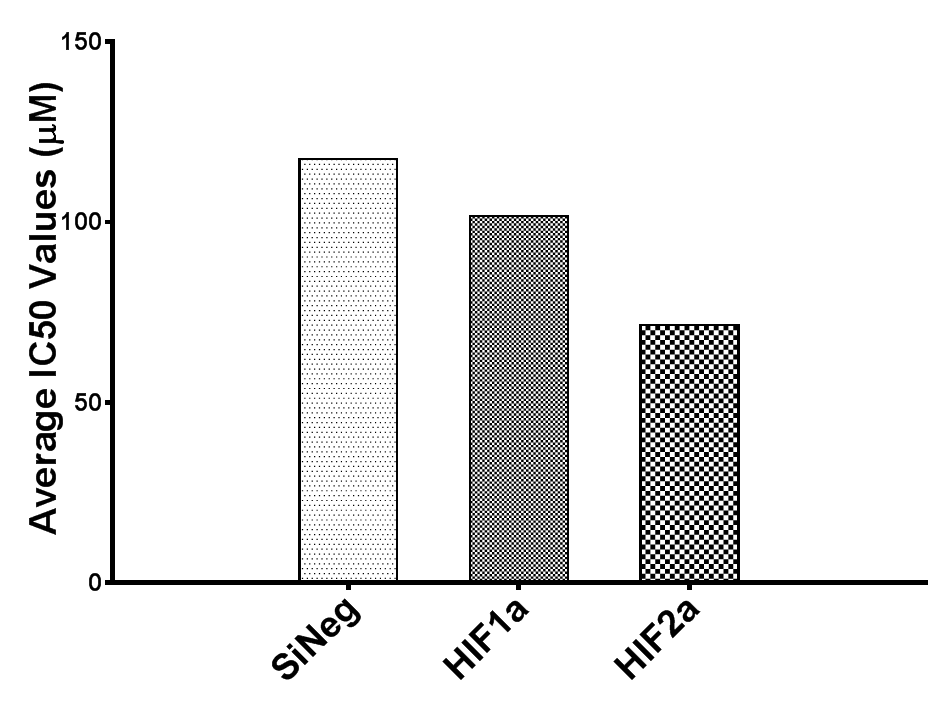


**Supp Fig. 4: HIFs sensitise U87 cells to cisplatin**. Following the downregulation of HIF1a and HIF2a in U87 cells, the mean IC_50_ values (µM) of cisplatin was evaluated based on an AlamarBlue assay. N=1


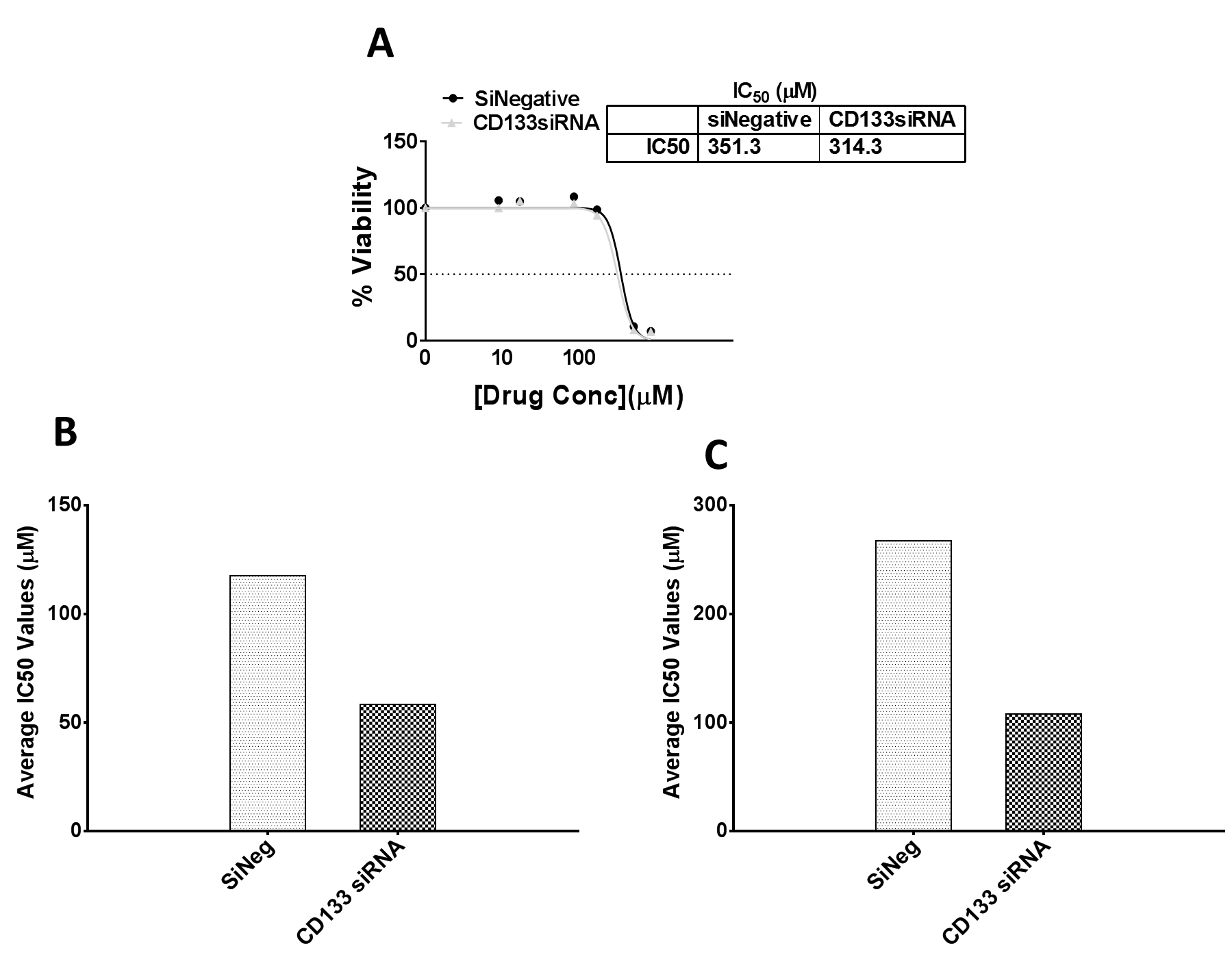


**Supp Fig 5: Effect of CD133 downregulation on temozolomide and cisplatin: (A) Downregulation of CD133 has no significant impact on temozolomide.** Following downregulation of CD133, U87 cells were treated with temozolomide and viability ascertained with AlamarBlue assay. **(B-C) CD133 dowregulation sensitizes glioblastoma cells to cisplatin.** Following downregulation of CD133, U87 (B) and SNB19 (C) cells were treated with cisplatin and viability ascertained with AlamarBlue assay. N=1
